# Identification and characterization of a gene conferring stress resistance in *Escherichia coli* and cyanobacteria

**DOI:** 10.1002/2211-5463.70343

**Published:** 2026-09-15

**Authors:** Akiyo Yamada, Takaaki Tanaka, Manaka Uehara, Keigo Nakano, Ken Morishima, Rintaro Inoue, Masaaki Sugiyama, Masafumi Yohda

**Affiliations:** ^1^ Department of Biotechnology and Life Science Tokyo University of Agriculture and Technology Fuchu Japan; ^2^ Institute for Integrated Radiation and Nuclear Science Kyoto University Japan

**Keywords:** analytical ultracentrifugation, chaperone, cyanobacteria, functional screening, small‐angle X‐ray scattering, stress

## Abstract

*Picosynechococcus* sp. strain NKBG15041c is a cyanobacterium isolated from the ocean and while it is known for rapid growth, the basis of its high proliferation capacity remains unclear. Screening for genes which may underlie this rapid growth using *E. coli*, we identified a gene that promotes growth and enhances stress resistance. The protein encoded by this gene (PcSyn14890) is specific to cyanobacteria, and no homologs with known functions have been identified. In *P*. NKBG15041c, expression of this gene was induced during the early stage of growth and when introduced into a different cyanobacterium, the transformants exhibited accelerated growth, enhanced stress resistance, and increased ATP and Chlorophyll *a* content. We hypothesized that PcSyn14890 functions as an ATP‐independent chaperone. To investigate further, recombinant PcSyn14890 was expressed in *E. coli*, purified, and subjected to structural and functional characterization. PcSyn14890 existed as monomers or dimers and did not form higher‐order oligomers. Its small‐angle X‐ray scattering profile was consistent with the AlphaFold3 predicted structure, and the CD spectrum confirmed its β‐sheet‐rich composition. However, contrary to expectations, PcSyn14890 was unable to prevent the thermal aggregation of glyceraldehyde‐3‐phosphate dehydrogenase (GAPDH). Thus, we conclude that PcSyn14890 does not function as a general molecular chaperone and therefore the mechanism by which expression of PcSyn14890 enhances the stress tolerance of *E. coli* and *S*. PCC6803 requires further investigation.

## Graphical abstract

Functional screening identified PcSyn14890, a cyanobacteria‐specific protein that enhances growth and stress resistance in *E. coli* and *Synechocystis* PCC6803. Although we expected it to function as a molecular chaperone, it was unable to protect against thermal aggregation of GAPDH. Further studies are needed to elucidate its mechanism of stress tolerance.

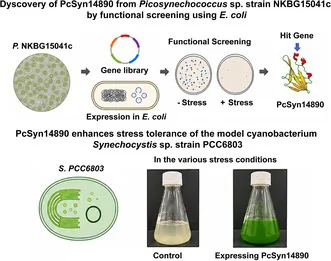

**Abbreviations**
AUC: analytical ultracentrifugation
GAPDH: glyceraldehyde‐3‐phosphate dehydrogenase
SAXS: small‐angle X‐ray scattering
sHsp: small heat shock protein

Cyanobacteria are autotrophic, gram‐negative bacteria belonging to the phylum *Cyanobacteriota* that generate energy through oxygenic photosynthesis [1]. They are considered the first organisms on Earth to have produced oxygen. The chloroplasts of modern plants are thought to have originated from cyanobacteria through endosymbiosis. As cyanobacteria contribute approximately 30% of the Earth's primary production, they play a crucial role in the global carbon cycle [2]. They are attracting increasing attention as production hosts for biofuels [3]. In addition to biofuels, they are being explored as platforms for the biosynthesis of valuable compounds such as fructose [4], mannitol [5], and high‐value pigments such as β‐carotene, lutein, and zeaxanthin [6], as well as biodegradable plastics such as polylactic acid and polyhydroxybutyrate (PHB) [7, 8]. Therefore, cyanobacteria are expected to play a significant role in addressing the challenges associated with worsening global warming. However, large‐scale practical applications have yet to be realized because of limitations in productivity. Cyanobacteria thrive in diverse environments and are exposed to various stresses, including fluctuations in light intensity, temperature, pH, nutrient availability, and the presence of toxic substances. To cope with these conditions, they have evolved a range of adaptive mechanisms, such as modulating gene expression, reconfiguring metabolic pathways, and producing protective compounds. Therefore, enhancing the stress resistance of cyanobacteria is essential for the efficient production of biofuels and biomaterials.

The marine cyanobacterium *Picosynechococcus* sp. strain NKBG15041c, previously named *Synechococcus* sp. strain NKBG15041c, is a strain isolated from the coastal waters of Okinawa Prefecture, Japan [9]. Compared with the model cyanobacterium *Synechocystis* sp. strain PCC6803, it has been reported to grow faster. Moreover, compared with *S*. PCC6803, this strain is significantly more resistant to thermal and acid stress. The complete genome sequence of *P*. NKBG15041c was recently determined [10]. The expression of a heterologous metallothionein gene enhances resistance to heavy metal ions [11], and the introduction of an alkane biosynthesis pathway enables the production of target compounds [12]. However, the underlying mechanisms responsible for the high growth capacity of this strain remain unclear. Elucidating these mechanisms could provide valuable insights for improving the growth performance of cyanobacteria in biotechnological applications.

In our laboratory, genes related to salt tolerance and growth have been identified from halophytic plants such as *Suaeda japonica* and mangroves and applied to microorganisms or higher plants using a “functional screening method with *Escherichia coli*” (PCT/JP00/04862) [13]. Using this method, we isolated various genes associated with stress resistance from a cDNA library of *P*. NKBG15041c. One clone exhibiting enhanced acid tolerance was found to contain a gene encoding a MoxR AAA+ ATPase, designated SyMRP [14]. The recombinant SyMRP protein displayed weak ATPase activity and was capable of protecting citrate synthase from thermal aggregation. Notably, the chaperone activity of SyMRP was ATP dependent. SyMRP existed as a stable hexamer, and no ATP‐dependent conformational changes were detected by analytical ultracentrifugation (AUC) or small‐angle X‐ray scattering (SAXS).

In the present study, we analyzed another gene and its product that confer stress resistance to *E. coli* and the model cyanobacterium *S*. PCC6803. The protein encoded by this gene, PcSyn14890, is specific to cyanobacteria, and no homologs with known functions have been identified. We performed structural and functional characterization of recombinant PcSyn14890. However, the mechanism by which PcSyn14890 enhances stress tolerance requires further investigation.

## Methods

### Strains and media

*P*. NKBG15041c and *S*. PCC6803 were provided by Prof. Tsuyoshi Tanaka of Tokyo University of Agriculture and Technology. *P*. NKBG15041c was cultured in BG11 medium supplemented with 3% (w/v) NaCl. *S*. PCC6803 was grown in standard BG11 medium.

### Genomic DNA library construction

Genomic DNA from *P*. NKBG15041c was extracted by bead beating with TE‐saturated phenol and glass beads, followed by phenol removal with chloroform, RNase treatment, and ethanol precipitation. The DNA was partially digested with Sau3AI, and fragments of 3–8 kb were isolated and ligated into the BamHI site of pBluescript SK(−). The resulting library was subsequently transformed into *E. coli* DH5α cells.

### Identification of salt tolerance‐enhancing genes using a functional screening method with *E. coli*

*E. coli* DH5α competent cells were transformed using the gene library, plated on 2xYT agar supplemented with 50 μg/mL ampicillin and 0.6 m NaCl, and then incubated at 37 °C for 15 h. Clones with strong growth were selected, and plasmids were extracted. For secondary screening, plasmids were retransformed into *E. coli* DH5α cells, which were subsequently plated on 2xYT Amp agar and incubated similarly. Selected clones were cultured until OD600 was 4.0–7.0, diluted, and spotted onto 2xYT Amp agar supplemented with 0.8 m NaCl. After 15 to 20 h at 37 °C, a clone with enhanced salt tolerance was identified and sequenced.

### Cloning of each gene in the selected clone

The three ORFs (NKBG15041C_14870, NKBG15041C_14880, and NKBG15041C_14890) contained in the obtained clone were amplified by PCR, digested with EcoRI and XhoI, and cloned and inserted into the EcoRI/XhoI sites of the pBluescript SK(−) vector. Primer sequences are shown in the Table S1. The proteins encoded by these ORFs are named PcSyn14870, PcSyn14880, and PcSyn14890, respectively.

### Spot test

*E. coli* cells transformed with each plasmid were precultured in 1.5 mL 2xYT Amp medium until OD_600_ reached 4.0–7.0. The culture was then diluted to an initial cell density of OD_600_ = 0.1. Afterward, 10 μL of the adjusted culture was spotted onto 2xYT Amp agar plates containing 0.9 m NaCl. The plates were incubated at 37 °C for approximately 15 to 20 h.

### Growth measurement of transformed *E. coli* under stress conditions

Transformed *E. coli* cells were adjusted to an initial cell density of OD_600_ = 0.1 and cultured in 2xYT Amp under the following conditions: 37 °C (control, nonstress) and 45 °C (heat stress) in medium containing 1.3 m sorbitol (osmotic stress) or 1.0 m NaCl (salt stress). The cultures were grown in L‐shaped test tubes with 4 mL of medium in each tube, with three replicates per condition.

### Expression pattern of NKBG15041C_14890 mRNA in *P.* NKBG15041c

*P*. NKBG15041c was cultured in BG11 medium supplemented with 0.5 m NaCl until the optical density at OD₇₃₀ reached 5.0–8.0 and then diluted to an initial OD₇₃₀ of 0.1 in 50 mL BG11 supplemented with 0.5 m NaCl. Cultures were incubated at 25 °C with shaking at 70 rpm and 45 μmol photons m^−2^·s^−1^ light. Algal cells were harvested on Days 0, 4, 12, and 28 and lyophilized, and approximately 5 mg was used for total RNA extraction using NucleoSpin RNA kit (Takara Bio, Shiga, Japan). cDNA was synthesized from 500 ng RNA using PrimeScript Reverse Transcriptase (Takara Bio). qPCR was performed with THUNDERBIRD SYBR qPCR Mix (Takara Bio) using primers for NKBG15041C14890 and 16S rRNA (see Table S1). Cycling conditions were 95 °C for 30 s, 58 °C for 30 s, and 72 °C for 30 s for 35 cycles.

### Construction of plasmids for expression in *S.* PCC6803

First, the gene was amplified using the primers PcSyn14890Fw1 and PcSyn14890Rv1. The amplified DNA was digested with NdeI and HindIII and then cloned and inserted into the NdeI/HindIII site of the pKTev vector, which was constructed by incorporating the C‐phycocyanin promoter, a multicloning site, and a terminator from *Synechococcus* into pKT230 [9]. The inserted gene was amplified by PCR using the primers PcSyn14890Fw2 and PcSyn14890Rv2 and then cloned and inserted into the AvrII/XhoI site of pKT‐Pcpc‐alkane [12]. The constructed plasmid was named pKT‐Pcpc‐PcSyn14890. Primer sequences are shown in the Table S1.

### Transformation of *S.* PCC6803

Electroporation was used for the transformation of cyanobacteria following a previously described method [15]. Cells of *S*. PCC6803 at OD_730_ = 0.5–1.0 were washed with HEPES buffer and resuspended in 100 μL. 60 μL of this suspension was mixed with 0.1–10 μg plasmid and electroporated (2400 V, 7.0 ms). After recovery in BG11 liquid medium for 5 days, cells were plated on BG11 agar supplemented with sodium thiosulfate and streptomycin. Transformants were selected after 2 weeks, and PcSyn14890 expression was confirmed.

### Growth of transformed cyanobacteria

The plasmids pKT‐Pcpc‐PcSyn14890 and pKT‐Pcpc (empty) were introduced into *S*. PCC6803 to generate transformants. These cyanobacterial cells were cultured in BG11/Sm liquid medium until OD₇₃₀ reached 5.0–8.0. The cultures were then diluted in 50 mL BG11/Sm medium to adjust the cell suspension to OD_730_ = 0.1. In the absence of stress, it was cultured in conditions of shaking at 32 °C, 45 μmol photons m^−2^·s^−1^, and 120 rpm. Growth curves were generated by measuring the absorbance at A₇₃₀ over time. The plants were cultivated under various stress conditions, such as salt stress (0.5 m NaCl), high light (200 μmol photons m^−2^·s^−1^), and high temperature (40 °C). For dry weight measurement, cells were harvested by centrifugation (20 400× ***g***, 5 min), freeze‐dried, and weighed.

### Measurement of chlorophyll *a* content

Chlorophyll *a* content and dry weight were also measured as indicators of growth. Cells were harvested by centrifugation (20 400× ***g***, 5 min), mixed with 1 mL of 100% methanol, and stirred. After standing at room temperature for 5 min, the mixture was centrifuged (9100 × g, 10 min), and the supernatant was collected. Absorbance at 665 nm (A_665_) was measured, and chlorophyll *a* concentration was calculated using the following formula [16]:

Chl *a* (μg/mL) = 13.4 × A_665_/(volume of harvested culture in mL).

### Measurement of ATP content

Intracellular ATP levels were measured using a BacTiter‐Glo Microbial Cell Viability Assay Kit (Promega, Madison, WI) according to the manufacturer's protocol. Luminescence was detected using a microplate reader (SH‐9000, CORONA ELECTRIC Co., Ibaraki, Japan). Intracellular ATP concentrations of each sample were calculated on the basis of a standard curve generated from measurements of ATP standards.

### Expression and purification of PcSyn14890

PcSyn14890 gene was amplified by PCR using the primers PcSyn14890_InFusion1 and PcSyn14890_InFusion2 and then cloned and inserted into the NdeI/Xho site of the pColdI vector (pColdI‐PcSyn14890) by In‐fusion method [17]. PcSyn14890 with a His‐tag at the C‐terminus was expressed in *E. coli* DH5α transformed with pCold‐PcSyn14890. PcSyn14890 was purified by affinity chromatography using Ni Sepharose™ 6 Fast Flow (Cytiva, Marlborough, MA); after dialysis, it was further purified by anion exchange chromatography (Whatman DE52, Sigma–Aldrich, St. Louis, MO, USA).

### Protein aggregation measurements

Thermal aggregation of glyceraldehyde‐3‐phosphate dehydrogenase from rabbit muscle (GAPDH; Sigma–Aldrich, St. Louis, MO) (8 μm) was assayed in 100 μL PBS at 45 °C. PcSyn14890 was added at 4, 8, 16, or 24 μm (final). Kinetics were recorded at 45 °C by static light scattering at 360 nm using CLARIOstar multimode plate reader (BMG LABTECH, Ortenberg, Germany) with one measurement every 30 s for 60 min; for each measurement, five consecutive shots were acquired and averaged by the instrument to smooth signal fluctuations. Baseline correction: For each well, the mean A360 over the first 5 min (10 reads at 30‐s intervals) was subtracted from the entire trace. Each condition was measured in triplicate wells (technical replicates, *n* = 3).

### Analytical ultracentrifugation (AUC) measurements

AUC measurements were carried out using a ProteomeLab XL‐I (Beckman Coulter, Brea, CA, USA). A 0.5 mg/mL PcSyn14890 solution was prepared in 50 mm Tris/HCl buffer (pH 8.0) containing 200 mm NaCl. The sample was loaded into a cell with a 12 mm optical path length double‐sector centerpiece. The measurements were carried out using Rayleigh interference optics at a rotor speed of 40 000 rpm and a temperature of 25 °C. The time evolution of the sedimentation data was analyzed using the multicomponent Lamm formula via SEDFIT software (version 15.01c) [18]. The weight concentration distribution *c*(*s*_20,w_) was subsequently obtained as a function of the sedimentation coefficient *s*_20,w_, which was normalized to the value at 20 °C in pure water. The molecular mass *M* of each component was calculated using the following equation:

(1) $$M={\left[\frac{6\pi \eta N_{A}}{\left(1-\rho \bar{v}\right)}\right]}^{1.5}{\left(\frac{3\bar{v}}{4\pi N_{A}}\right)}^{0.5}{\left(\frac{f}{f_{0}}\right)}^{1.5}{s_{20,w}}^{1.5},$$

where ρ, η, $\bar{v}$, $N_{A}$, and *f*/*f*_0_ are the density of pure water at 20 °C, viscosity of pure water at 20 °C, partial specific volume, Avogadro number, and frictional ratio, respectively.

### Small‐angle X‐ray scattering (SAXS) measurements

SAXS measurements were carried out using a laboratory‐based instrument (NANOPIX; Rigaku, Tokyo, Japan) for a 0.5 mg/mL PcSyn14890 solution in 50 mm Tris/HCl buffer (pH 8.0) containing 200 mm NaCl with and without 1 mm ATP. Prior to the SAXS measurement, the solution was centrifuged at 17 000 ***g*** and 4 °C for 10 min. The solution was subsequently filtered by a spin filter with a pore size of 0.22 μm. Point‐focused X‐rays at a wavelength of 1.54 Å were generated by a Cu K source MicroMAX‐007 HFMR (Rigaku). Scattered X‐rays were measured with a HyPix‐6000 hybrid photon counting detector (Rigaku). The sample‐to‐detector distances were set to 1330 mm and 300 mm, covering a *q* range of 0.01 Å^−1^ ≤ *q* ≤ 0.70 Å^−1^, where *q* is the magnitude of the scattering vector. Two‐dimensional scattering patterns were converted to one‐dimensional scattering profiles using SAngler software [19]. The measurement was carried out at 25 °C.

Since the coexistence of oligomers and monomers was detected by AUC measurement, AUC‐SAXS treatment was applied to derive the scattering profile of PcSyn14890 monomer from the SAXS profile. Details of the AUC‐SAXS treatment have been described elsewhere [20, 21].

### Molecular modeling

Molecular modeling of PcSyn14890 monomer was performed using AlphaFold3 [22]. The SAXS profile from the molecular model was calculated via CRYSOL 3.0 in the ATSAS software package [23]. The agreement between the calculated SAXS profile *I*_cal_(*q*) and the experimental profile *I*_exp_(*q*) was evaluated using the $\chi^{2}$ value as follows.

(2) $$\chi^{2}=\frac{1}{N-1}\sum_{i}^{N}{\left[\frac{I_{\exp}\left(q_{i}\right)-{\mathrm{bI}}_{\mathrm{cal}}\left(q_{i}\right)}{\sigma \left(q_{i}\right)}\right]}^{2},$$

where *N* is the number of points in the scattering profile, σ(*q*) are the experimental errors, and *b* is the scaling factor as follows.

(3) $$b=\left[\sum_{i}^{N}\frac{I_{\exp}\left(q_{i}\right)I_{\mathrm{cal}}\left(q_{i}\right)}{\sigma {\left(q_{i}\right)}^{2}}\right]/\left[\sum_{i}^{N}\frac{I_{\mathrm{cal}}{\left(q_{i}\right)}^{2}}{\sigma {\left(q_{i}\right)}^{2}}\right].$$

### Far‐UV circular dichroism spectroscopy

CD spectrum of 13.9 μm PcSyn14890 in 50 mm Tris/Cl pH 8.0, 150 mm NaCl was recorded on a J‐720 spectropolarimeter (JASCO) using a quartz cell with a 1 mm optical path length. The scanning speed was 500 nm/min with a response time of 1 s, and the wavelength range was 180–250 nm.

## Results

### Identification of the gene that confers resistance to stress in *E. coli*

By functional screening, we identified one clone that conferred significant salt stress resistance to *E. coli*. The inserted DNA contained three ORFs, which were named c32.03, c32.04, and c32.05. After the completion of genome sequencing, they were designated NKBG15041C_14870, NKBG15041C_14880, and NKBG15041C_14890, respectively. The encoded proteins showed no homology to any known functional proteins and are conserved only in *Picosynechococcus* spp. Hereafter, we name the proteins PcSyn14870, PcSyn14880, and PcSyn14890, respectively.

**Fig. 1:**
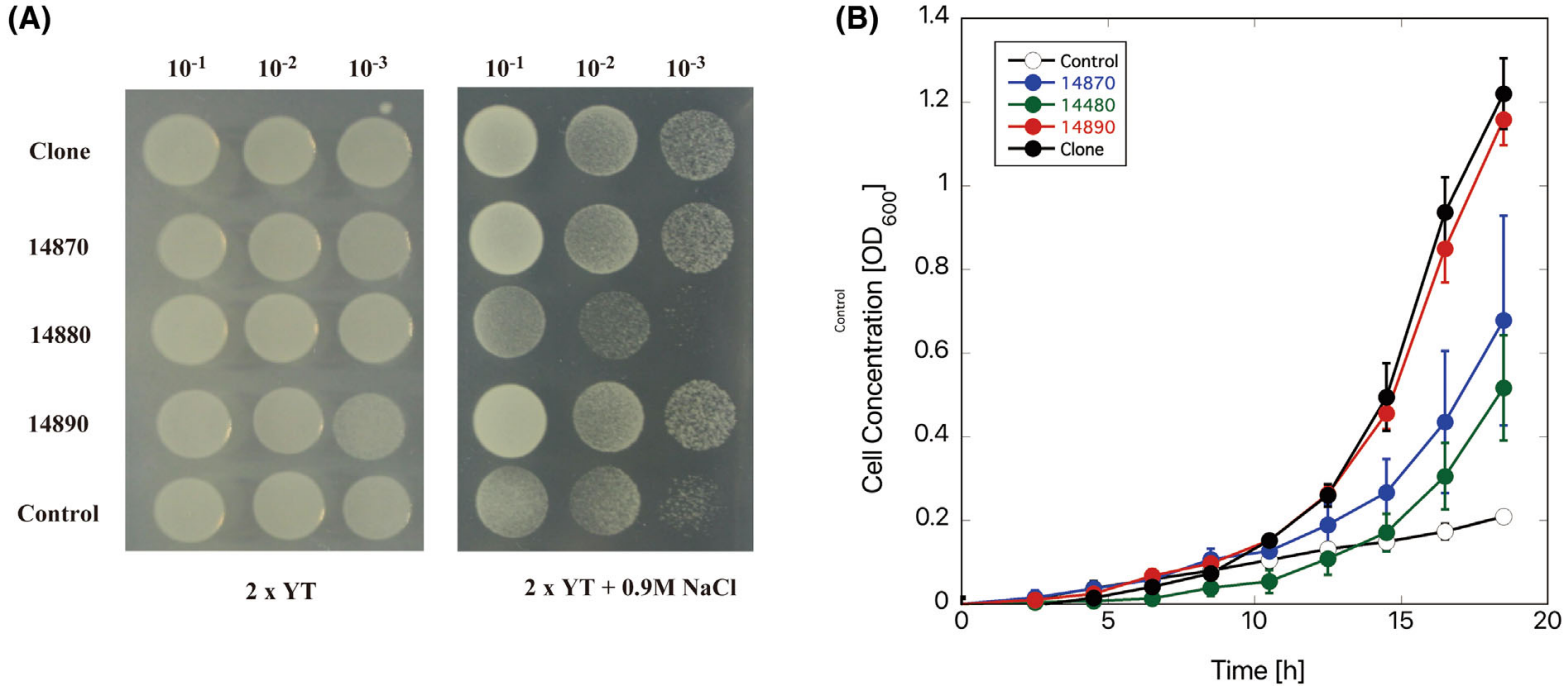
Screening of a gene that confers salt and osmotic resistance to *E. coli*. (A) Spot tests for the salt tolerance of *E. coli* transformants. (B) Evaluation of osmotic tolerance of *E. coli* transformants. Black: Original Clone, Blue: NKBG15041C_14870, Green: NKBG15041C_14880, Red: NKBG15041C_14890. The error bars indicate the standard deviation of three independent experiments (SD).

Initially, we attempted to identify the gene responsible for the stress response. Each gene was amplified by PCR, cloned, and inserted into the pBluescriptSK(−) vector. We then compared the stress tolerance of *E. coli* transformants carrying the plasmids. In a spot assay, *E. coli* strains transformed with plasmids containing PcSyn14870‐ or PcSyn14890‐encoding genes exhibited salt tolerance comparable to that of the original clone (Fig. 1A). We subsequently evaluated osmotic tolerance using 2xYT liquid medium supplemented with 1.3 m sorbitol (Fig. 1B). While the control strain showed little to no growth, *E. coli* transformants harboring genes derived from *P*. NKBG15041c demonstrated enhanced growth. Notably, the strains carrying either the original clone or the plasmid containing the PcSyn14890‐encoding gene exhibited particularly robust growth. On the basis of these results, we selected PcSyn14890 for further study.

**Fig. 2:**
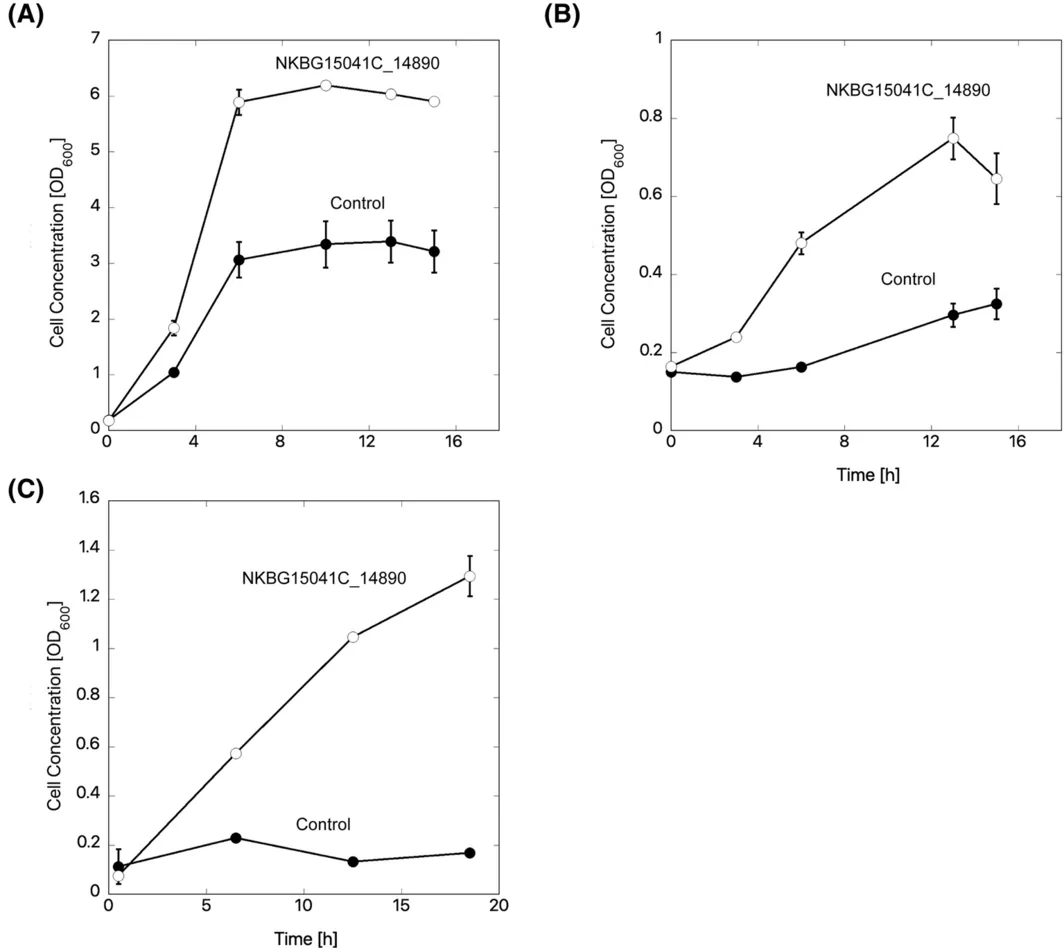
Growth curves of *E. coli* transformants in 2YT medium under nonstress conditions. (A), salt stress (1.0 m NaCl) (B) and heat stress (45 °C) (C). Open circle NKBG15041C_14890, closed circle control vector. The error bars indicate the standard deviation of three independent experiments (SD).

Compared with the control strain, *E. coli* expressing PcSyn14890 grew significantly faster (Fig. 2A). In addition, it exhibited resistance to both salt and heat stress (Fig. 2B and C).

**Fig. 3:**
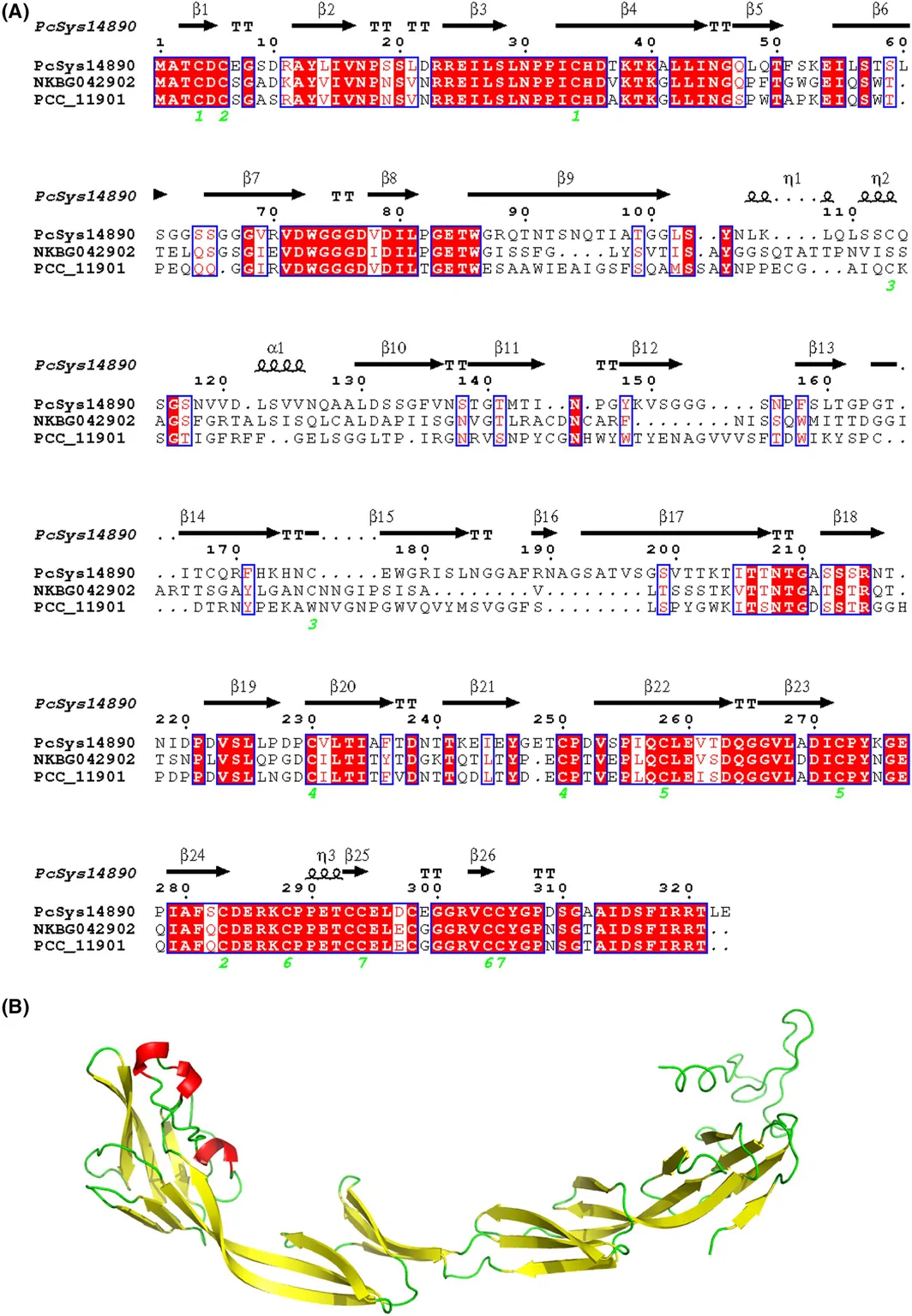
Sequence alignment and predicted structure of PcSyn14890. (A) Sequence alignment and secondary structure. Sequence alignment was performed using ClustalW. (B) Predicted structure of PcSyn14890. The predicted structure was generated using AlphaFold3, and the image was rendered using pymol.

Sequence alignment with homologs in other *Picosynechococcus* spp. and predicted model structure of PySyn14890 are shown in Fig. 3. PySyn14890 has an extended structure with high β‐strand propensity.

### Evaluation of PcSyn14890‐encoding gene expression pattern in *P.* NKBG15041c

**Fig. 4:**
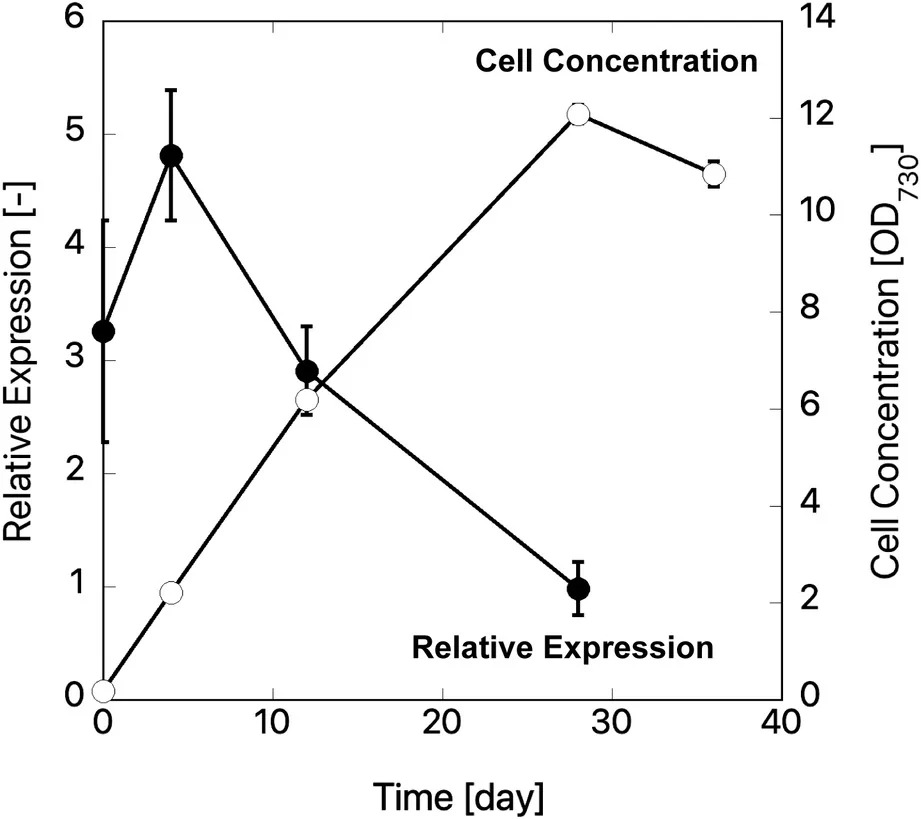
Expression analysis of NKBG15041C_14890 in *P*. NKBG15041c cells. The open circle indicates the relative expression of NKBG15041C_14890. Closed circle indicates the growth of *P*. NKBG15041c. The error bars indicate the standard deviation of three independent experiments (SD).

To analyze the expression pattern of PcSyn14890‐encoding gene in *P*. NKBG15041c, quantitative PCR was performed to measure PcSyn14890 mRNA expression at different growth stages. The results revealed that the expression of the PcSyn14890‐encoding gene was greatest during the active growth phase and gradually decreased as growth slowed (Fig. 4).

### Phenotypic analysis of transformed cyanobacteria

**Fig. 5:**
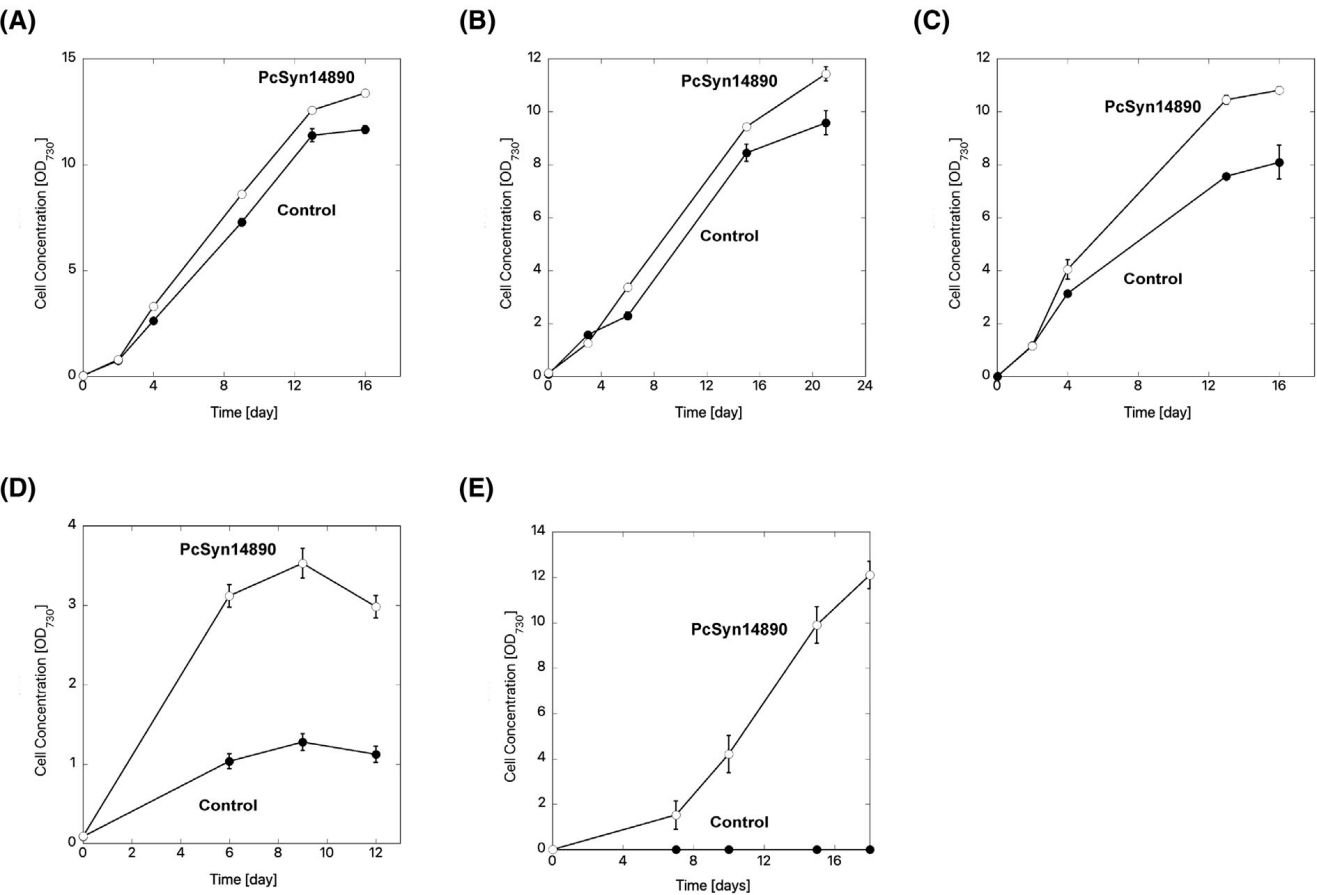
Growth curves of *S*. PCC6803 transformants in BG11 medium under nonstress (A), salt stress (0.5 m NaCl) (B), heat stress (40 °C) (C), high light (200 μmol photons m^−2^ s^−1^) (D), and oxidative stress (0.5 mm H_2_O_2_) (E) conditions. Open and closed circles indicate growth of the transformant expressing PcSyn14890 and the vector control, respectively. The error bars indicate the standard deviation of three independent experiments (SD).

Next, we investigated the effect of PcSyn14890‐encoding gene on the stress tolerance of cyanobacteria. PcSyn14890‐encoding gene was inserted into a cyanobacterial expression vector and introduced into *S*. PCC6803. The differences in growth between the *S*. PCC6803 strain expressing PcSyn14890 and the control strain harboring the empty vector were evaluated under both nonstress conditions and various stress conditions. Under nonstress conditions, the growth capacity of the PcSyn14890‐expressing strain was greater than that of the control, with its dry weight after 16 days of cultivation reaching approximately 1.26 times that of the control (Fig. 5A). Quantification of chlorophyll *a* revealed that compared with the control strain, the PcSyn14890‐expressing strain contained a higher level of chlorophyll *a* (Table 1). The difference of the relative content was statistically significant (*t* = 18.5, *n* = 3, *P* = 0.000341). In addition, compared with the control strain, the PcSyn14890‐expressing strain presented approximately three times higher intracellular ATP levels (Table 2). The difference of the relative content was also statistically significant (*t* = 11.2, *n* = 3, *P* = 0.00153).

**Table 1:** Dry cell weight and chlorophyll a content of S. PCC6803 transformants. Transformants were precultured in BG11 medium until they reached an A730 of 5.0–8.0 and then inoculated into fresh BG11 at an initial A730 of 0.1. Cultures were grown at 32 °C with agitation at 120 rpm for 16 days. Dry cell weight and chlorophyll *a* content were measured. Data are presented as means ± SEs of three independent experiments.

	Dry cell weight (g/L)	Chlorophyll *a* (mg/L)	Relative content (mg/g)
Control	1.9 ± 0.1	11.7 ± 0.2	6.15 ± 0.1
PcSyn14890	2.4 ± 0.1	33.0 ± 0.9	13.8 ± 0.4

**Table 2:** Intracellular ATP contents of S. PCC6803 transformants under nonstress conditions. Transformants were precultured in BG11 medium. The transformants were grown to 6.0–8.0 (A730), cells were inoculated in BG11 medium, and the initial cell concentration was adjusted to 0.1 (A730). Cultures were grown at 32 °C. The liquid cultures were agitated at 120 rpm. Intracellular ATP contents of transformants cultured for 16 days were measured. Data are presented as means ± SEs of three independent experiments.

	Control	PcSyn14890
ATP contents (*n* mol/g DCW)	67.3 ± 4.5	192.9 ± 10.3

Growth under salt, high light, heat stress and oxidative stress conditions was also examined. In all stress conditions tested, differences in growth were observed between the PcSyn14890‐expressing strain and the control, indicating enhanced stress tolerance (Fig. 5 BCDE). Notably, significant differences in growth were observed under high light and oxidative stress conditions.

### Expression, purification and structural characterization of recombinant PcSyn14890

**Fig. 6:**
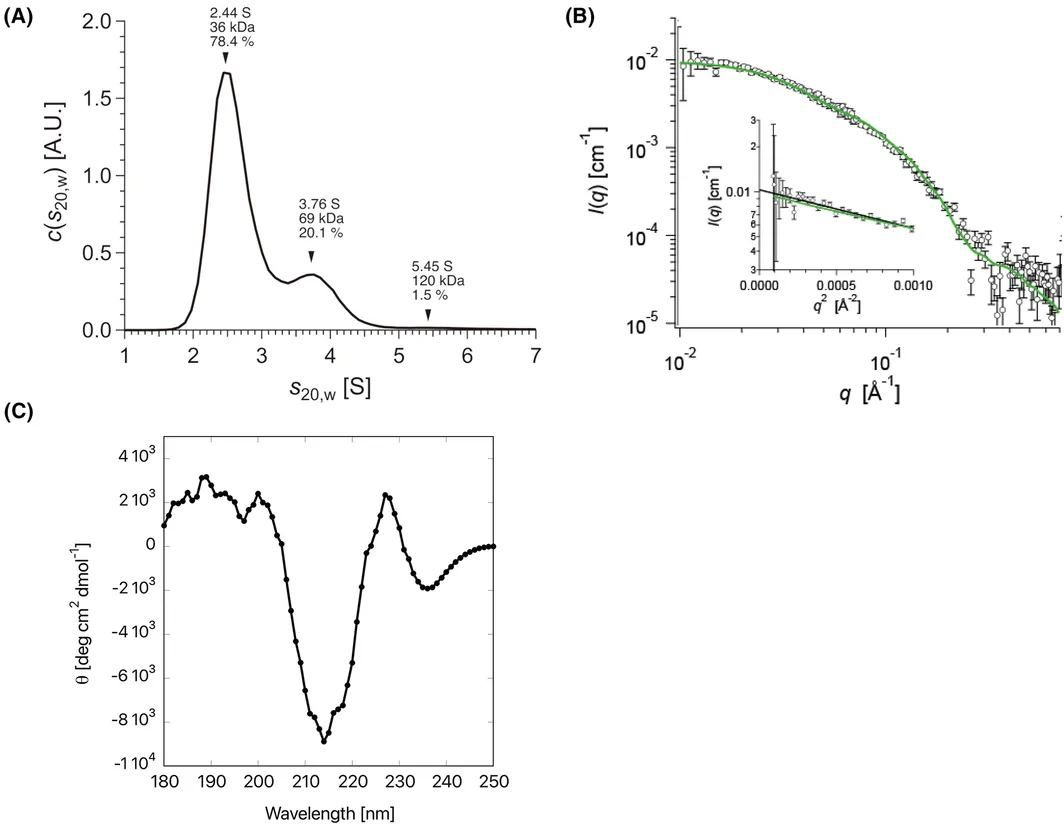
Structural characterization of PcSyn14890. (A) Analytical ultracentrifugation (AUC) results for PcSyn14890 solution. Weight concentration distribution *c*(*s*_20,w_) was obtained as a function of the sedimentation coefficient *s*_20,w_, which was normalized to the value at 20 °C in pure water. Sedimentation coefficient, molecular mass, and weight fraction for each component are noted. (B) Small‐angle X‐ray scattering (SAXS) profile for PcSyn14890 solution. Open circles represent SAXS profile of PcSyn14890 monomer, which was derived via AUC‐SAXS treatment. Solid green line indicates the scattering profile calculated for the structural model (Fig. 3B). Inset represents their Guinier plot. Solid black line indicates the least square fitting line with Guinier formula. Data are presented as the means ± SDs of three independent experiments. (C) Far‐UV Circular Dichroism Spectroscopy. CD spectrum of PcSyn14890 in a quartz cell with a 1 mm optical path length. The scanning speed was 500 nm/min with a response time of 1 s, and the wavelength range was 180–250 nm.

PcSyn14890 with His‐tag at the C terminus was expressed using pCold I vector. PcSyn14890 was purified by affinity chromatography and anion exchange chromatography. Analytical ultracentrifugation (AUC) analysis revealed separation into two peaks, with calculated molecular weights of 36 kDa and 69 kDa, which correspond to monomer and dimer (Fig. 6A). The SAXS profile of PcSyn14890 monomer, which was derived by AUC‐SAXS treatment, is shown in Fig. 6B [20, 21]. The molecular mass calculated from the forward scattering intensity was 30 ± 2 kDa, which was approximately consistent with that of PcSyn14890 monomer. The gyration radius *R*_g_ determined from Guinier analysis was 42.2 ± 1.8 Å [24]. The SAXS profile matched well with the profile calculated from the model structure, as shown in Fig. 6B ($\chi^{2}$ = 2.2), suggesting that the extended structure with high β‐strand propensity is a reasonable model in the solution. In the CD spectrum of PcSyn14890, a negative maximum at 214 nm appeared, which is the characteristic of β‐sheet secondary structure (Fig. 6C).

### Effect of PcSyn14890 on thermal aggregation of GAPDH

**Fig. 7:**
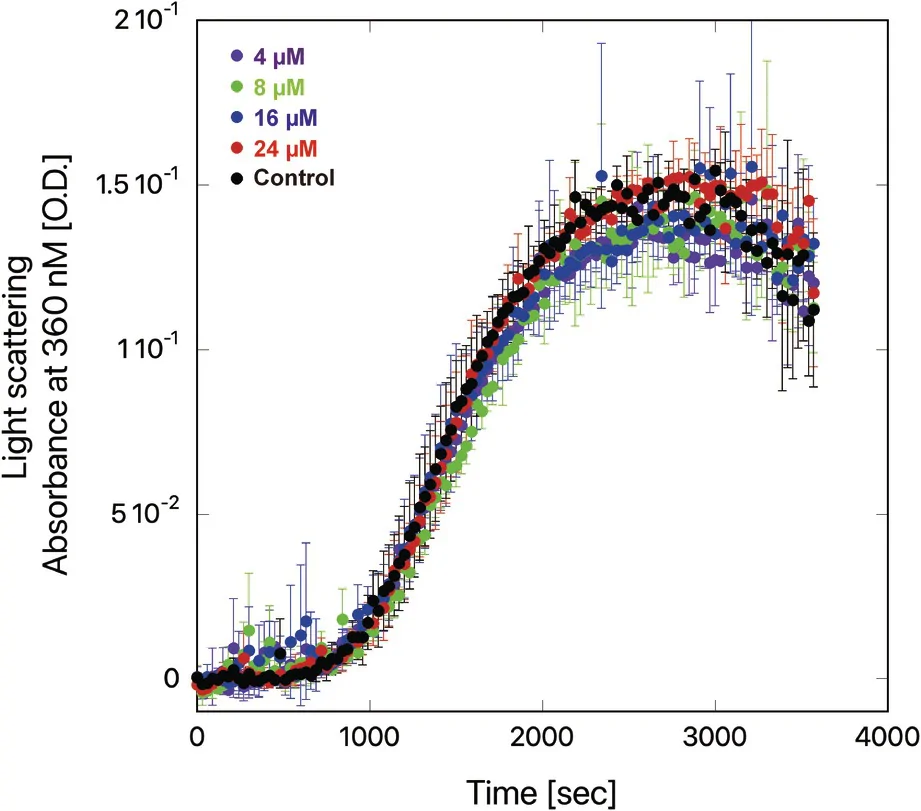
Effect of PcSyn14890 on thermal aggregation of glyceraldehyde‐3‐phosphate dehydrogenase (GAPDH). GAPDH (8 μm) was incubated at 45 °C in the absence (black) or presence of PcSyn14890 at final concentrations of 4 (purple), 8 (green), 16 (blue), or 24 (red) μm. The error bars represent the standard error of the mean (SEM) of three independent experiments.

Since the expression of PcSyn14890 enables cyanobacteria and *E. coli* to acquire various stress tolerances and takes a β‐sheet‐rich structure similar to sHsp, it was hypothesized that PcSyn14890 functions as a molecular chaperone. Therefore, the thermal aggregation suppression activity of PcSyn14890 on GAPDH was measured (Fig. 7). PcSyn14890 was unable to suppress the thermal aggregation of GAPDH.

## Discussion

We identified stress tolerance‐associated genes from *P*. NKBG15041C through functional screening using *E. coli*. The clones obtained in this study contained three genes (NKBG15041C_014870, NKBG15041C_014880, and NKBG15041C_014890). Since NKBG15041C_014880 is encoded on the complementary strand of other genes, these genes do not form operons. None of these genes share homology with known functional proteins and appear to encode cyanobacteria‐specific proteins. Among these, NKBG15041C_014870 and NKBG15041C_014890 individually conferred salt stress tolerance to *E. coli*. On the basis of a comparison of osmotic stress tolerance, we focused our investigation on NKBG15041C_014890. However, it is likely that the other genes, NKBG15041C_014870 and NKBG15041C_014880, might also contribute to the stress resistance of *P*. NKBG15041C.

*E. coli* cells introduced with NKBG15041_014890 exhibited improved growth compared to the control not only under salt and high‐temperature stress conditions but also under nonstress conditions. Even in the nonstress condition, cells may suffer from some stresses caused by increased cell density and the accumulation of waste products. Thus, the effect should also be due to the stress resistance induced by NKBG15041_014890. We hypothesized that PcSyn14890, encoded by NKBG15041_014890, may confer *E. coli* stress resistance by maintaining proteostasis as a molecular chaperone. This idea is partly supported by our previous results that a gene cloned using the same method encodes MoxR ATPase with molecular chaperone activity [14]. Moreover, a previous study demonstrated that overexpression of the chaperonin, groES and groEL, confers a cyanobacterium better growth than the wild type in both unstressed and stressed conditions [25].

PcSyn14890 lacks the ATPase active site found in various molecular chaperones, such as chaperonin, Hsp70, Hsp90, or Hsp100. Structural prediction by AlphaFold3 indicated that PcSyn14890 adopts a structure rich in β‐sheets. The SAXS profile calculated on the basis of this predicted structure matched the experimental data well, supporting the likelihood that PcSyn14890 adopts this conformation. Among various molecular chaperones, small heat shock proteins (sHsps) also take β‐sheet‐rich structure. They are ATP‐independent molecular chaperones that quickly bind unfolding proteins to maintain proteostasis until energy‐dependent chaperones such as chaperonins, Hsp70, and Hsp100 restore proper folding or promote degradation [26, 27]. Under normal conditions, sHsps form large oligomers of 12 to 36 subunits, which dissociate into smaller oligomers under stress, exposing hydrophobic surfaces that prevent protein aggregation. [28].

Expecting a similar functional mechanism, the oligomeric structure of PcSyn14890 was analyzed, revealing existence as monomer or dimer forms, exhibiting behavior different from that of typical sHsps. PcSyn14890 was unable to protect GAPDH from thermal aggregation. Thus, it is unlikely that the enhanced stress resistance is due to its general chaperone activity. Because of its extended structure, PcSyn14890 may interact with various proteins which may relate to proteostasis.

NKBG15041C_014890 is most highly expressed during the early growth phase of *P*. NKBG15041C. Since *P*. NKBG15041C is known for its rapid growth, these findings suggest that NKBG15041C_014890 may play a role in its high growth rate. In support of this hypothesis, the expression of NKBG15041C_014890 in cyanobacteria led to increased growth, increased biomass production, elevated chlorophyll *a* content, and improved stress tolerance.

The mechanism by which PcSyn14890 expression increases chlorophyll *a* levels remains unknown. Because PcSyn14890 is also effective in *E. coli*, it is unlikely that PcSyn14890 directly contributes to chlorophyll a biosynthesis. Instead, the effect may result from improved proteostasis within the cell. Since PcSyn14890 itself is not a general molecular chaperone, it is reasonable to assume that its expression alters the intracellular proteome, thereby indirectly promoting chlorophyll *a* accumulation.

## Conclusion

By a functional screening method, we discovered a gene that promotes *E. coli* growth and enhances stress resistance. The protein encoded by this gene, PcSyn14890, is specific to cyanobacteria. PcSyn14890 is a cyanobacteria‐specific protein that, when introduced into *S*. PCC6803, boosts growth and stress resistance. Although its structure resembles an ATP‐independent molecular chaperone, sHsp, experiments showed it does not function as a general chaperone. The mechanism by which expression of PcSyn14890 enhances the stress tolerance of *E. coli* and *S*. PCC6803 requires further investigation.

## Conflicts of interest

The authors declare no conflicts of interest.

## Author contributions

**Akiyo Yamada:** resources, project administration, funding acquisition, validation, methodology, investigation, writing – original draft, writing – review and editing. **Takaaki Tanaka:** Investigation. **Manaka Uehara:** investigation. **Keigo Nakano:** investigation. **Ken Morishima:** visualization, validation, methodology, investigation, formal analysis, data curation. **Rintaro Inoue:** visualization, validation, methodology, investigation, formal analysis, data curation. **Masaaki Sugiyama:** visualization, validation, methodology, investigation, formal analysis, data curation, funding acquisition. **Masafumi Yohda:** writing – review and editing, writing – original draft, visualization, validation, supervision, methodology, investigation, funding acquisition, formal analysis, data curation.

## Supporting information


**Table S1.** Sequences of primers used in this study.

## Data Availability

All data of this article are available from the corresponding author upon request.
