# Supplementary material for: Identification and characterization of a gene conferring stress resistance in Escherichia coli and cyanobacteria
Source: FEBS Open Bio. 2026 Sep 15:10.1002/2211-5463.70343. Online ahead of print. doi: 10.1002/2211-5463.70343 (PMC13576663; doi:10.1002/2211-5463.70343)
Supplement: Supplementary file 1 [file FEB4-9999-0-s001.docx]

**Supplementary Table**

**Sequences of primers used in this study**

| **Primers for cloning** | |
| --- | --- |
| NKBG15041C_14870 Forward | 5'-CGCCGAATTCAAGAGTAAAAGCCATTAATC-3' |
| NKBG15041C_14870 Reverse | 5'-CTTCTCGAGCTATGAGCTGTCCCAATCTTG-3' |
| NKBG15041C_14880 Forward | 5'-TCAGAATTCGTCGCAAGATTGGGACAGCTC-3' |
| NKBG15041C_14880 Reverse | 5'-CCGCTCGAGACTTGCTGTGAGCTTGACTGC-3' |
| NKBG15041C_14890 Forward | 5'-TTAGAATTCCGCCCGATAAACTCCCACCAA-3' |
| NKBG15041C_14890 Reverse | 5'-AGGCTCGAGAACAATTGTTAAGAGAAAGGG-3' |
| **Primers used for qPCR** | |
| NKBG15041C_14890_RT_Fw | 5'-GGGACGATCACCTGTCAGAG-3' |
| NKBG15041C_14890_RT_Rv | 5'-GAGCGGCCTGATTAACGACA-3' |
| 16SrRNA_RT_Fw | 5'-CACACTGGGACTGAGACAC-3' |
| 16SrRNA_RT_Rv | 5'-CTGCTGGCACGGAGTTAG-3' |
| **Primers for the expression in *Synechocystis* sp. PCC6803** | |
| PcSys14890Fw1 | 5'-TTGCATATGAAGGGGATAGAGAACTGATGG-3' |
| PcSys14890Rv1 | 5'-GTCAAGCTTTCACTCATTTAAGTTCTCCTG-3' |
| PcSys14890Fw2 | 5'-TTGcctaggAAGGGGATAGAGAACTGATGG-3' |
| PcSys14890Rv2 | 5'-TTTctcgagCTCAGGAGAGCGTTCACCGAC-3' |
| **Primers for the expression in *E. coli*** | |
| PcSyn14890_InFusion1 | 5’-ATCGAAGGTAGGCATATGGCTACTTGTGATTGTGAG-3’ |
| PcSyn14890_InFusion2 | 5’-ATTCGGATCCCTCGAGTTAAGTTCTCCTGATAAAAGAAT-3’ |
